# Supplementary material for: Modeling glioblastoma heterogeneity as a dynamic network of cell states
Source: Mol Syst Biol. 2021 Sep 16;17(9):e10105. doi: 10.15252/msb.202010105 (PMC8444284; doi:10.15252/msb.202010105)
Supplement: Supplementary file 6 — Source Data for Figure 5 [file MSB-17-e10105-s004.zip › Figure5A_sourcedata/GSEA_3071/nosigdb_stateVI.GseaPreranked.1622795169056/RICHARDS_NOWAKOWSKI_SCIENCE_2017_MICROGLIA_UPREG.html]

Details for gene set RICHARDS\_NOWAKOWSKI\_SCIENCE\_2017\_MICROGLIA\_UPREG[GSEA]

|  || Dataset | state63071 |
| Phenotype | NoPhenotypeAvailable |
| Upregulated in class | na\_pos |
| GeneSet | RICHARDS\_NOWAKOWSKI\_SCIENCE\_2017\_MICROGLIA\_UPREG |
| Enrichment Score (ES) | 0.17814791 |
| Normalized Enrichment Score (NES) | 1.590796 |
| Nominal p-value | 0.0 |
| FDR q-value | 0.049167283 |
| FWER p-Value | 0.473 |
Table: GSEA Results Summary

  

Fig 1: Enrichment plot: RICHARDS\_NOWAKOWSKI\_SCIENCE\_2017\_MICROGLIA\_UPREG      
 Profile of the Running ES Score & Positions of GeneSet Members on the Rank Ordered List

  

| PROBE | GENE SYMBOL | GENE\_TITLE | RANK IN GENE LIST | RANK METRIC SCORE | RUNNING ES | CORE ENRICHMENT || 1 | HMGA1 |  |  | 1 | 1.500 | 0.0088 | Yes |
| 2 | CITED2 |  |  | 5 | 1.232 | 0.0155 | Yes |
| 3 | DDIT3 |  |  | 14 | 1.086 | 0.0197 | Yes |
| 4 | AXL |  |  | 33 | 0.946 | 0.0200 | Yes |
| 5 | PLAUR |  |  | 34 | 0.936 | 0.0258 | Yes |
| 6 | ODC1 |  |  | 38 | 0.918 | 0.0305 | Yes |
| 7 | ETS2 |  |  | 52 | 0.864 | 0.0318 | Yes |
| 8 | SNHG12 |  |  | 59 | 0.851 | 0.0352 | Yes |
| 9 | COTL1 |  |  | 60 | 0.851 | 0.0404 | Yes |
| 10 | ABHD12 |  |  | 74 | 0.799 | 0.0414 | Yes |
| 11 | DAB2 |  |  | 78 | 0.788 | 0.0453 | Yes |
| 12 | GLIPR1 |  |  | 81 | 0.781 | 0.0494 | Yes |
| 13 | LPXN |  |  | 85 | 0.772 | 0.0532 | Yes |
| 14 | GADD45B |  |  | 90 | 0.758 | 0.0566 | Yes |
| 15 | ZFAND2A |  |  | 95 | 0.739 | 0.0599 | Yes |
| 16 | SNAPC1 |  |  | 96 | 0.738 | 0.0645 | Yes |
| 17 | CPED1 |  |  | 97 | 0.738 | 0.0690 | Yes |
| 18 | PPP1R15A |  |  | 98 | 0.738 | 0.0735 | Yes |
| 19 | C12orf75 |  |  | 99 | 0.738 | 0.0780 | Yes |
| 20 | POU2F2 |  |  | 109 | 0.725 | 0.0797 | Yes |
| 21 | GAS6 |  |  | 113 | 0.719 | 0.0832 | Yes |
| 22 | MIR22HG |  |  | 133 | 0.686 | 0.0816 | Yes |
| 23 | TAF1D |  |  | 135 | 0.685 | 0.0855 | Yes |
| 24 | DUSP1 |  |  | 136 | 0.685 | 0.0897 | Yes |
| 25 | EIF5 |  |  | 138 | 0.683 | 0.0936 | Yes |
| 26 | SQSTM1 |  |  | 147 | 0.667 | 0.0952 | Yes |
| 27 | CAP1 |  |  | 150 | 0.666 | 0.0987 | Yes |
| 28 | ELL2 |  |  | 156 | 0.655 | 0.1012 | Yes |
| 29 | MYOF |  |  | 157 | 0.655 | 0.1052 | Yes |
| 30 | DUSP5 |  |  | 169 | 0.631 | 0.1057 | Yes |
| 31 | HBEGF |  |  | 172 | 0.627 | 0.1089 | Yes |
| 32 | MYL12A |  |  | 173 | 0.627 | 0.1127 | Yes |
| 33 | SERPINB1 |  |  | 184 | 0.618 | 0.1135 | Yes |
| 34 | NABP1 |  |  | 186 | 0.609 | 0.1169 | Yes |
| 35 | CHCHD7 |  |  | 198 | 0.604 | 0.1173 | Yes |
| 36 | RNASET2 |  |  | 226 | 0.584 | 0.1128 | Yes |
| 37 | TNFAIP2 |  |  | 244 | 0.568 | 0.1111 | Yes |
| 38 | TACC1 |  |  | 245 | 0.568 | 0.1146 | Yes |
| 39 | MAFF |  |  | 253 | 0.562 | 0.1159 | Yes |
| 40 | PMAIP1 |  |  | 255 | 0.561 | 0.1190 | Yes |
| 41 | VASP |  |  | 257 | 0.561 | 0.1221 | Yes |
| 42 | CLIC1 |  |  | 258 | 0.561 | 0.1256 | Yes |
| 43 | SH3BGRL3 |  |  | 265 | 0.556 | 0.1271 | Yes |
| 44 | RIPK2 |  |  | 267 | 0.555 | 0.1302 | Yes |
| 45 | FKBP4 |  |  | 279 | 0.550 | 0.1303 | Yes |
| 46 | TWISTNB |  |  | 280 | 0.549 | 0.1336 | Yes |
| 47 | ZFP36L2 |  |  | 284 | 0.547 | 0.1361 | Yes |
| 48 | SP100 |  |  | 294 | 0.544 | 0.1367 | Yes |
| 49 | MYH9 |  |  | 312 | 0.534 | 0.1348 | Yes |
| 50 | JUN |  |  | 321 | 0.527 | 0.1356 | Yes |
| 51 | HSPB1 |  |  | 322 | 0.527 | 0.1389 | Yes |
| 52 | LGALS1 |  |  | 323 | 0.526 | 0.1421 | Yes |
| 53 | MKNK2 |  |  | 326 | 0.524 | 0.1447 | Yes |
| 54 | GLRX |  |  | 330 | 0.523 | 0.1469 | Yes |
| 55 | USP53 |  |  | 340 | 0.515 | 0.1474 | Yes |
| 56 | PRR13 |  |  | 345 | 0.514 | 0.1493 | Yes |
| 57 | RGS10 |  |  | 346 | 0.513 | 0.1524 | Yes |
| 58 | PFN1 |  |  | 347 | 0.513 | 0.1556 | Yes |
| 59 | EIF5A |  |  | 361 | 0.507 | 0.1548 | Yes |
| 60 | SERTAD1 |  |  | 380 | 0.496 | 0.1524 | Yes |
| 61 | NR3C1 |  |  | 395 | 0.492 | 0.1512 | Yes |
| 62 | ATF5 |  |  | 401 | 0.490 | 0.1527 | Yes |
| 63 | TXNIP |  |  | 402 | 0.487 | 0.1556 | Yes |
| 64 | RELB |  |  | 406 | 0.486 | 0.1577 | Yes |
| 65 | HSPA5 |  |  | 414 | 0.484 | 0.1585 | Yes |
| 66 | ATF3 |  |  | 416 | 0.483 | 0.1612 | Yes |
| 67 | C6orf48 |  |  | 427 | 0.479 | 0.1611 | Yes |
| 68 | TNFAIP3 |  |  | 445 | 0.473 | 0.1589 | Yes |
| 69 | CD55 |  |  | 452 | 0.471 | 0.1600 | Yes |
| 70 | USP36 |  |  | 453 | 0.470 | 0.1628 | Yes |
| 71 | CTSB |  |  | 471 | 0.464 | 0.1605 | Yes |
| 72 | CDV3 |  |  | 476 | 0.462 | 0.1622 | Yes |
| 73 | SNHG5 |  |  | 478 | 0.461 | 0.1647 | Yes |
| 74 | ACTR3 |  |  | 486 | 0.459 | 0.1654 | Yes |
| 75 | WDR74 |  |  | 504 | 0.454 | 0.1630 | Yes |
| 76 | KLF10 |  |  | 506 | 0.453 | 0.1655 | Yes |
| 77 | DDX21 |  |  | 511 | 0.452 | 0.1671 | Yes |
| 78 | PLA2G16 |  |  | 519 | 0.450 | 0.1677 | Yes |
| 79 | RPL35 |  |  | 525 | 0.449 | 0.1689 | Yes |
| 80 | AMD1 |  |  | 537 | 0.444 | 0.1683 | Yes |
| 81 | NANS |  |  | 543 | 0.443 | 0.1695 | Yes |
| 82 | MAP2K3 |  |  | 550 | 0.440 | 0.1704 | Yes |
| 83 | SLC3A2 |  |  | 559 | 0.437 | 0.1707 | Yes |
| 84 | EIF1 |  |  | 568 | 0.434 | 0.1709 | Yes |
| 85 | CD68 |  |  | 570 | 0.433 | 0.1733 | Yes |
| 86 | BHLHE41 |  |  | 573 | 0.432 | 0.1753 | Yes |
| 87 | DNAJB4 |  |  | 578 | 0.431 | 0.1767 | Yes |
| 88 | SERPINB6 |  |  | 583 | 0.430 | 0.1781 | Yes |
| 89 | OSER1 |  |  | 597 | 0.426 | 0.1768 | No |
| 90 | PIK3CD |  |  | 615 | 0.421 | 0.1743 | No |
| 91 | PNP |  |  | 625 | 0.418 | 0.1741 | No |
| 92 | DAGLB |  |  | 632 | 0.416 | 0.1749 | No |
| 93 | PABPC4 |  |  | 633 | 0.415 | 0.1774 | No |
| 94 | TBC1D4 |  |  | 652 | 0.411 | 0.1745 | No |
| 95 | TPD52L2 |  |  | 653 | 0.411 | 0.1770 | No |
| 96 | ARPC2 |  |  | 673 | 0.406 | 0.1738 | No |
| 97 | RBMS1 |  |  | 678 | 0.405 | 0.1751 | No |
| 98 | LIMS1 |  |  | 697 | 0.398 | 0.1721 | No |
| 99 | ARL4A |  |  | 704 | 0.395 | 0.1727 | No |
| 100 | ETF1 |  |  | 715 | 0.393 | 0.1721 | No |
| 101 | OLFML3 |  |  | 721 | 0.392 | 0.1730 | No |
| 102 | ABCC4 |  |  | 737 | 0.389 | 0.1708 | No |
| 103 | TCP1 |  |  | 758 | 0.384 | 0.1672 | No |
| 104 | CTSL |  |  | 767 | 0.383 | 0.1671 | No |
| 105 | RNF149 |  |  | 780 | 0.380 | 0.1658 | No |
| 106 | RAP1A |  |  | 782 | 0.380 | 0.1678 | No |
| 107 | PTP4A2 |  |  | 790 | 0.379 | 0.1680 | No |
| 108 | KLF2 |  |  | 814 | 0.374 | 0.1634 | No |
| 109 | FLOT1 |  |  | 827 | 0.372 | 0.1621 | No |
| 110 | SLC25A3 |  |  | 828 | 0.372 | 0.1643 | No |
| 111 | HERPUD1 |  |  | 830 | 0.371 | 0.1663 | No |
| 112 | BIN1 |  |  | 836 | 0.371 | 0.1671 | No |
| 113 | RNF135 |  |  | 842 | 0.369 | 0.1678 | No |
| 114 | ARPC1B |  |  | 847 | 0.369 | 0.1689 | No |
| 115 | CAPG |  |  | 851 | 0.368 | 0.1702 | No |
| 116 | KLF6 |  |  | 854 | 0.368 | 0.1719 | No |
| 117 | YBX3 |  |  | 855 | 0.368 | 0.1741 | No |
| 118 | DOCK10 |  |  | 858 | 0.367 | 0.1757 | No |
| 119 | GSTO1 |  |  | 871 | 0.365 | 0.1744 | No |
| 120 | JAG1 |  |  | 876 | 0.365 | 0.1754 | No |
| 121 | SHC1 |  |  | 913 | 0.358 | 0.1668 | No |
| 122 | TPM3 |  |  | 915 | 0.358 | 0.1686 | No |
| 123 | DDX39A |  |  | 936 | 0.355 | 0.1648 | No |
| 124 | IER5 |  |  | 950 | 0.353 | 0.1630 | No |
| 125 | RPL13 |  |  | 966 | 0.350 | 0.1607 | No |
| 126 | TPI1 |  |  | 972 | 0.350 | 0.1613 | No |
| 127 | TLN1 |  |  | 976 | 0.349 | 0.1625 | No |
| 128 | PPIF |  |  | 983 | 0.348 | 0.1629 | No |
| 129 | NOP10 |  |  | 995 | 0.347 | 0.1617 | No |
| 130 | RRBP1 |  |  | 999 | 0.346 | 0.1629 | No |
| 131 | ABL2 |  |  | 1033 | 0.339 | 0.1550 | No |
| 132 | BAG3 |  |  | 1034 | 0.339 | 0.1571 | No |
| 133 | NEU1 |  |  | 1053 | 0.337 | 0.1538 | No |
| 134 | CAST |  |  | 1070 | 0.334 | 0.1510 | No |
| 135 | IFRD1 |  |  | 1085 | 0.333 | 0.1488 | No |
| 136 | HSPA9 |  |  | 1087 | 0.333 | 0.1505 | No |
| 137 | HSP90AA1 |  |  | 1088 | 0.333 | 0.1526 | No |
| 138 | CAPZA1 |  |  | 1111 | 0.329 | 0.1480 | No |
| 139 | PICALM |  |  | 1116 | 0.328 | 0.1488 | No |
| 140 | PSMA6 |  |  | 1117 | 0.327 | 0.1508 | No |
| 141 | CEBPZOS |  |  | 1124 | 0.327 | 0.1510 | No |
| 142 | ARPC3 |  |  | 1139 | 0.325 | 0.1487 | No |
| 143 | RHBDF2 |  |  | 1151 | 0.323 | 0.1474 | No |
| 144 | SLC25A5 |  |  | 1163 | 0.321 | 0.1460 | No |
| 145 | RRP12 |  |  | 1217 | 0.313 | 0.1320 | No |
| 146 | EVI2A |  |  | 1221 | 0.313 | 0.1330 | No |
| 147 | TNS3 |  |  | 1232 | 0.311 | 0.1319 | No |
| 148 | ACTB |  |  | 1243 | 0.310 | 0.1308 | No |
| 149 | B3GNT5 |  |  | 1308 | 0.302 | 0.1135 | No |
| 150 | PITPNA |  |  | 1329 | 0.300 | 0.1093 | No |
| 151 | EPB41L2 |  |  | 1343 | 0.299 | 0.1072 | No |
| 152 | RHOG |  |  | 1355 | 0.297 | 0.1057 | No |
| 153 | UBE2D3 |  |  | 1361 | 0.296 | 0.1060 | No |
| 154 | VDAC1 |  |  | 1362 | 0.296 | 0.1078 | No |
| 155 | TLN2 |  |  | 1383 | 0.293 | 0.1036 | No |
| 156 | CEBPB |  |  | 1389 | 0.293 | 0.1039 | No |
| 157 | HIF1A |  |  | 1458 | 0.284 | 0.0852 | No |
| 158 | RAP1B |  |  | 1467 | 0.283 | 0.0845 | No |
| 159 | RPL22L1 |  |  | 1473 | 0.283 | 0.0847 | No |
| 160 | HSP90AB1 |  |  | 1492 | 0.281 | 0.0810 | No |
| 161 | MTO1 |  |  | 1514 | 0.278 | 0.0764 | No |
| 162 | MTCH1 |  |  | 1539 | 0.275 | 0.0709 | No |
| 163 | HSPD1 |  |  | 1556 | 0.274 | 0.0678 | No |
| 164 | HEXB |  |  | 1568 | 0.272 | 0.0661 | No |
| 165 | JMJD6 |  |  | 1580 | 0.271 | 0.0645 | No |
| 166 | ARID3A |  |  | 1639 | 0.265 | 0.0487 | No |
| 167 | LRIF1 |  |  | 1643 | 0.265 | 0.0494 | No |
| 168 | MGAT1 |  |  | 1649 | 0.264 | 0.0495 | No |
| 169 | PLCB2 |  |  | 1655 | 0.264 | 0.0496 | No |
| 170 | NAA20 |  |  | 1680 | 0.262 | 0.0440 | No |
| 171 | PLK3 |  |  | 1710 | 0.259 | 0.0369 | No |
| 172 | CHKA |  |  | 1733 | 0.257 | 0.0318 | No |
| 173 | CTSC |  |  | 1767 | 0.254 | 0.0235 | No |
| 174 | TBXAS1 |  |  | 1779 | 0.253 | 0.0217 | No |
| 175 | RPL36A |  |  | 1788 | 0.252 | 0.0208 | No |
| 176 | TAPBP |  |  | 1817 | -0.252 | 0.0140 | No |
| 177 | CNPY3 |  |  | 1851 | -0.257 | 0.0056 | No |
| 178 | ADAM17 |  |  | 1855 | -0.258 | 0.0063 | No |
| 179 | STAT3 |  |  | 1861 | -0.259 | 0.0064 | No |
| 180 | CTSD |  |  | 1862 | -0.259 | 0.0079 | No |
| 181 | NCK2 |  |  | 1865 | -0.260 | 0.0089 | No |
| 182 | PDIA3 |  |  | 1879 | -0.261 | 0.0066 | No |
| 183 | FOXO1 |  |  | 1929 | -0.269 | -0.0065 | No |
| 184 | NAIP |  |  | 1936 | -0.269 | -0.0066 | No |
| 185 | HIST1H3D |  |  | 1946 | -0.270 | -0.0077 | No |
| 186 | SAT1 |  |  | 1952 | -0.271 | -0.0075 | No |
| 187 | ATP6AP2 |  |  | 1974 | -0.274 | -0.0122 | No |
| 188 | FMNL3 |  |  | 1992 | -0.277 | -0.0156 | No |
| 189 | CPVL |  |  | 2000 | -0.278 | -0.0160 | No |
| 190 | CREM |  |  | 2019 | -0.282 | -0.0197 | No |
| 191 | TGOLN2 |  |  | 2055 | -0.288 | -0.0284 | No |
| 192 | GATM |  |  | 2082 | -0.292 | -0.0345 | No |
| 193 | ST3GAL6 |  |  | 2083 | -0.292 | -0.0327 | No |
| 194 | RASSF4 |  |  | 2123 | -0.301 | -0.0425 | No |
| 195 | NCOR2 |  |  | 2134 | -0.303 | -0.0437 | No |
| 196 | PARP14 |  |  | 2145 | -0.304 | -0.0449 | No |
| 197 | TPP1 |  |  | 2191 | -0.313 | -0.0565 | No |
| 198 | ZNF217 |  |  | 2192 | -0.313 | -0.0546 | No |
| 199 | NEAT1 |  |  | 2208 | -0.316 | -0.0571 | No |
| 200 | DNAJB11 |  |  | 2251 | -0.324 | -0.0678 | No |
| 201 | TMEM107 |  |  | 2271 | -0.327 | -0.0715 | No |
| 202 | ARHGAP12 |  |  | 2302 | -0.333 | -0.0785 | No |
| 203 | TIAM1 |  |  | 2303 | -0.334 | -0.0764 | No |
| 204 | SETX |  |  | 2307 | -0.334 | -0.0753 | No |
| 205 | CD276 |  |  | 2308 | -0.334 | -0.0732 | No |
| 206 | METTL7A |  |  | 2313 | -0.335 | -0.0724 | No |
| 207 | CDC42SE1 |  |  | 2358 | -0.346 | -0.0835 | No |
| 208 | RNF213 |  |  | 2383 | -0.350 | -0.0886 | No |
| 209 | ENPP2 |  |  | 2391 | -0.352 | -0.0885 | No |
| 210 | BAX |  |  | 2397 | -0.353 | -0.0879 | No |
| 211 | SIRPA |  |  | 2398 | -0.353 | -0.0857 | No |
| 212 | B2M |  |  | 2460 | -0.369 | -0.1018 | No |
| 213 | ITGAV |  |  | 2476 | -0.371 | -0.1041 | No |
| 214 | ABHD3 |  |  | 2487 | -0.375 | -0.1048 | No |
| 215 | VMP1 |  |  | 2490 | -0.376 | -0.1031 | No |
| 216 | ARID5A |  |  | 2496 | -0.378 | -0.1023 | No |
| 217 | DDX5 |  |  | 2507 | -0.379 | -0.1030 | No |
| 218 | ZNF148 |  |  | 2539 | -0.386 | -0.1099 | No |
| 219 | MMP2 |  |  | 2550 | -0.388 | -0.1106 | No |
| 220 | SPRY2 |  |  | 2569 | -0.391 | -0.1136 | No |
| 221 | NDE1 |  |  | 2588 | -0.397 | -0.1166 | No |
| 222 | SSH1 |  |  | 2596 | -0.400 | -0.1162 | No |
| 223 | RSRP1 |  |  | 2611 | -0.403 | -0.1180 | No |
| 224 | KCTD12 |  |  | 2629 | -0.407 | -0.1206 | No |
| 225 | PET100 |  |  | 2631 | -0.408 | -0.1184 | No |
| 226 | MAF |  |  | 2633 | -0.408 | -0.1162 | No |
| 227 | MEF2A |  |  | 2638 | -0.409 | -0.1149 | No |
| 228 | FNDC3B |  |  | 2645 | -0.410 | -0.1142 | No |
| 229 | CHSY1 |  |  | 2684 | -0.420 | -0.1231 | No |
| 230 | ANKRD44 |  |  | 2705 | -0.427 | -0.1265 | No |
| 231 | NFE2L2 |  |  | 2707 | -0.427 | -0.1242 | No |
| 232 | PAG1 |  |  | 2720 | -0.430 | -0.1252 | No |
| 233 | OS9 |  |  | 2724 | -0.431 | -0.1234 | No |
| 234 | PREX1 |  |  | 2727 | -0.432 | -0.1214 | No |
| 235 | ATP6AP1 |  |  | 2779 | -0.449 | -0.1340 | No |
| 236 | RAB31 |  |  | 2806 | -0.459 | -0.1390 | No |
| 237 | SRGAP2 |  |  | 2821 | -0.463 | -0.1404 | No |
| 238 | SPRED1 |  |  | 2825 | -0.464 | -0.1384 | No |
| 239 | FUS |  |  | 2845 | -0.474 | -0.1412 | No |
| 240 | LDLRAD4 |  |  | 2854 | -0.478 | -0.1407 | No |
| 241 | FTH1 |  |  | 2871 | -0.484 | -0.1426 | No |
| 242 | CFLAR |  |  | 2902 | -0.495 | -0.1486 | No |
| 243 | PLD3 |  |  | 2922 | -0.503 | -0.1512 | No |
| 244 | DNASE2 |  |  | 2924 | -0.503 | -0.1485 | No |
| 245 | PDE4B |  |  | 2948 | -0.513 | -0.1522 | No |
| 246 | GALNT1 |  |  | 2978 | -0.528 | -0.1577 | No |
| 247 | ARHGEF6 |  |  | 3003 | -0.544 | -0.1616 | No |
| 248 | GRN |  |  | 3016 | -0.547 | -0.1619 | No |
| 249 | TGFBR1 |  |  | 3017 | -0.548 | -0.1585 | No |
| 250 | ID2 |  |  | 3026 | -0.554 | -0.1576 | No |
| 251 | CHD1 |  |  | 3033 | -0.556 | -0.1560 | No |
| 252 | SIPA1L2 |  |  | 3038 | -0.557 | -0.1538 | No |
| 253 | GSN |  |  | 3041 | -0.559 | -0.1510 | No |
| 254 | TCEAL3 |  |  | 3051 | -0.564 | -0.1502 | No |
| 255 | ASAH1 |  |  | 3052 | -0.564 | -0.1468 | No |
| 256 | PNRC1 |  |  | 3055 | -0.566 | -0.1440 | No |
| 257 | CD81 |  |  | 3056 | -0.566 | -0.1405 | No |
| 258 | RNF13 |  |  | 3089 | -0.580 | -0.1466 | No |
| 259 | TNFRSF21 |  |  | 3125 | -0.600 | -0.1534 | No |
| 260 | MALT1 |  |  | 3128 | -0.605 | -0.1503 | No |
| 261 | WSB1 |  |  | 3130 | -0.608 | -0.1469 | No |
| 262 | MIS18BP1 |  |  | 3146 | -0.615 | -0.1477 | No |
| 263 | MYO9B |  |  | 3159 | -0.627 | -0.1475 | No |
| 264 | GYPC |  |  | 3169 | -0.638 | -0.1463 | No |
| 265 | PTPRJ |  |  | 3174 | -0.640 | -0.1436 | No |
| 266 | GNAI2 |  |  | 3183 | -0.647 | -0.1420 | No |
| 267 | ITM2C |  |  | 3187 | -0.652 | -0.1390 | No |
| 268 | SKIL |  |  | 3188 | -0.652 | -0.1350 | No |
| 269 | ETV6 |  |  | 3221 | -0.690 | -0.1404 | No |
| 270 | RB1 |  |  | 3229 | -0.697 | -0.1383 | No |
| 271 | ATP1B1 |  |  | 3245 | -0.711 | -0.1384 | No |
| 272 | PSAP |  |  | 3252 | -0.714 | -0.1359 | No |
| 273 | MCF2L |  |  | 3255 | -0.715 | -0.1321 | No |
| 274 | ADPGK |  |  | 3270 | -0.729 | -0.1319 | No |
| 275 | SALL1 |  |  | 3284 | -0.742 | -0.1313 | No |
| 276 | EGR1 |  |  | 3285 | -0.742 | -0.1267 | No |
| 277 | JUND |  |  | 3295 | -0.755 | -0.1248 | No |
| 278 | NFIC |  |  | 3296 | -0.756 | -0.1202 | No |
| 279 | RGS16 |  |  | 3304 | -0.765 | -0.1177 | No |
| 280 | GNG7 |  |  | 3310 | -0.774 | -0.1144 | No |
| 281 | HTRA1 |  |  | 3331 | -0.796 | -0.1156 | No |
| 282 | FCGRT |  |  | 3341 | -0.807 | -0.1134 | No |
| 283 | SRGAP2C |  |  | 3365 | -0.833 | -0.1152 | No |
| 284 | HEXA |  |  | 3370 | -0.840 | -0.1113 | No |
| 285 | CHST11 |  |  | 3371 | -0.840 | -0.1062 | No |
| 286 | SRGAP2B |  |  | 3372 | -0.841 | -0.1010 | No |
| 287 | BTG2 |  |  | 3377 | -0.857 | -0.0970 | No |
| 288 | GNB4 |  |  | 3390 | -0.874 | -0.0953 | No |
| 289 | IRF2BPL |  |  | 3402 | -0.895 | -0.0931 | No |
| 290 | MAML2 |  |  | 3441 | -0.983 | -0.0985 | No |
| 291 | APLP2 |  |  | 3446 | -1.001 | -0.0936 | No |
| 292 | SLC29A1 |  |  | 3449 | -1.005 | -0.0881 | No |
| 293 | SLC1A3 |  |  | 3458 | -1.025 | -0.0843 | No |
| 294 | ETV5 |  |  | 3466 | -1.043 | -0.0800 | No |
| 295 | FOS |  |  | 3476 | -1.056 | -0.0763 | No |
| 296 | LITAF |  |  | 3491 | -1.089 | -0.0738 | No |
| 297 | NINJ1 |  |  | 3500 | -1.117 | -0.0694 | No |
| 298 | A2M |  |  | 3503 | -1.122 | -0.0632 | No |
| 299 | DUSP6 |  |  | 3512 | -1.151 | -0.0586 | No |
| 300 | SPECC1 |  |  | 3523 | -1.202 | -0.0543 | No |
| 301 | ITM2B |  |  | 3540 | -1.269 | -0.0513 | No |
| 302 | SPP1 |  |  | 3541 | -1.286 | -0.0435 | No |
| 303 | IL1RAP |  |  | 3551 | -1.364 | -0.0379 | No |
| 304 | ITPR2 |  |  | 3556 | -1.388 | -0.0306 | No |
| 305 | SPRY1 |  |  | 3565 | -1.432 | -0.0243 | No |
| 306 | PDPN |  |  | 3580 | -1.590 | -0.0188 | No |
| 307 | GNAS |  |  | 3585 | -1.610 | -0.0102 | No |
| 308 | SORL1 |  |  | 3609 | -1.895 | -0.0055 | No |
| 309 | NRP2 |  |  | 3617 | -2.285 | 0.0063 | No |
Table: GSEA details [plain text format]

  

Fig 2: RICHARDS\_NOWAKOWSKI\_SCIENCE\_2017\_MICROGLIA\_UPREG: Random ES distribution      
 Gene set null distribution of ES for **RICHARDS\_NOWAKOWSKI\_SCIENCE\_2017\_MICROGLIA\_UPREG**
